# Epigenetic landscape of synthetic human centromere core regions: CENP-A assembly and euchromatic modifications interdependently antagonize heterochromatin accumulation

**DOI:** 10.1093/nar/gkag597

**Published:** 2026-06-30

**Authors:** Junichiro Ohzeki, Akiko Watanabe, Jia Xian Lee, Chiharu Minami, Kazuto Kugou, Kyotaro Yamazaki, Kenta Shirasawa, Sachiko Isobe, Yusuke Endo, Yasuhiro Kazuki

**Affiliations:** Chromosome Engineering Research Center, Tottori University, 86 Nishi-cho, Yonago, Tottori 683-8503, Japan; Chromosome Engineering Research Group, The Exploratory Research Center on Life and Living Systems (ExCELLS), National Institutes of Natural Sciences, 5-1 Higashiyama, Myodaiji, Okazaki, Aichi 444-8787, Japan; Homeostatic Regulation, National Institute for Physiological Sciences, National Institutes of Natural Sciences, 5-1 Higashiyama, Myodaiji, Okazaki 444-8787, Japan; Kazusa DNA Research Institute (KDRI), 2-6-7 Kazusa-Kamatari, Kisarazu, Chiba 292-0818, Japan; Division of Chromosome Biomedical Engineering, Graduate School of Medical Science, Tottori University, 86 Nishi-cho, Yonago, Tottori 683-8503, Japan; Kazusa DNA Research Institute (KDRI), 2-6-7 Kazusa-Kamatari, Kisarazu, Chiba 292-0818, Japan; Kazusa DNA Research Institute (KDRI), 2-6-7 Kazusa-Kamatari, Kisarazu, Chiba 292-0818, Japan; Chromosome Engineering Research Group, The Exploratory Research Center on Life and Living Systems (ExCELLS), National Institutes of Natural Sciences, 5-1 Higashiyama, Myodaiji, Okazaki, Aichi 444-8787, Japan; Homeostatic Regulation, National Institute for Physiological Sciences, National Institutes of Natural Sciences, 5-1 Higashiyama, Myodaiji, Okazaki 444-8787, Japan; Kazusa DNA Research Institute (KDRI), 2-6-7 Kazusa-Kamatari, Kisarazu, Chiba 292-0818, Japan; Kazusa DNA Research Institute (KDRI), 2-6-7 Kazusa-Kamatari, Kisarazu, Chiba 292-0818, Japan; Kazusa DNA Research Institute (KDRI), 2-6-7 Kazusa-Kamatari, Kisarazu, Chiba 292-0818, Japan; Chromosome Engineering Research Center, Tottori University, 86 Nishi-cho, Yonago, Tottori 683-8503, Japan; Chromosome Engineering Research Group, The Exploratory Research Center on Life and Living Systems (ExCELLS), National Institutes of Natural Sciences, 5-1 Higashiyama, Myodaiji, Okazaki, Aichi 444-8787, Japan; Division of Chromosome Biomedical Engineering, Graduate School of Medical Science, Tottori University, 86 Nishi-cho, Yonago, Tottori 683-8503, Japan; Department of Chromosome Biomedical Engineering, School of Life Science, Faculty of Medicine, Tottori University, 86 Nishi-cho, Yonago, Tottori 683-8503, Japan

## Abstract

Centromeres are specialized chromatin structures essential for equal chromosome segregation. The human centromeres are organized on a portion of homogeneously repeated DNA sequence and contains nucleosomes including centromere specific histone H3 variant CENP-A. Histone modifications around the CENP-A dense region are also thought to have important roles in centromere function. Here, we used human artificial chromosome (HAC) system based on synthetic centromeric repeat DNA to enable structural and chromatin analysis at ∼2-kb resolution within defined centromere core domains. Analysis using this HAC centromere showed that the CENP-A dense regions span approximately 18–50 kb in size and coexisted with euchromatic histone modifications in an interdependent manner. We further found that when DNA replication reduced CENP-A density, a heterochromatin modification H3K9me3 transiently accumulated in the CENP-A-dense regions. This accumulation was suppressed by the CENP-A deposition factor HJURP and the histone acetyltransferase KAT7, suggesting that CENP-A assembly and euchromatic modifications interdependently antagonize heterochromatin accumulation. The synthetic centromere DNA generated in this study elucidates the epigenetic landscape within the centromere core regions and provides a more precise framework for understanding the dynamic balance between CENP-A assembly and histone modifications.

## Graphical Abstract

**Graphical Abstract:**
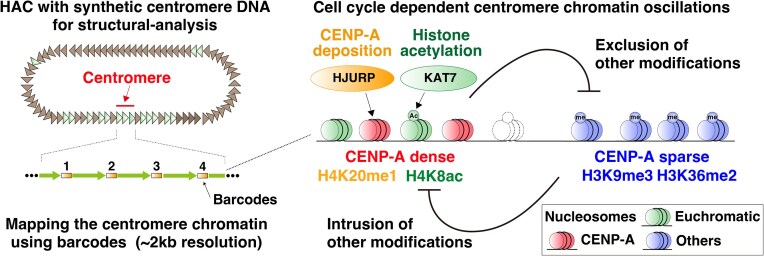

## Introduction

Genomic information is maintained as chromosomes. Centromere is essential and specialized chromatin structure for equal chromosome segregation. Centromere chromatin contains specific histone H3 variant, CENP-A [1, 2]. CENP-A forms nucleosome with histone H2A, H2B, and H4 and interacts with other constitutive centromere-associated network (CCAN) protein components [3–11]. During mitotic phase, kinetochore proteins assemble onto the centromere chromatin, and equally distribute sister chromosomes through interaction with spindle microtubules and checkpoint proteins [9, 12–15]. Once the CCAN proteins assembly is established, centromeric CENP-A nucleosome assembly is epigenetically maintained by CENP-A replenishing factors. In human cells, CENP-A amounts per centromere are reduced by DNA replication but are not returned during replicative or mitotic phase. CENP-A replenishing factors target centromeric chromatin after telophase when kinetochore proteins dissociate, and return CENP-A amounts during early G1 phase [16–21]. The centromere, kinetochore, and associated epigenetic maintenance factors and functions are largely conserved in vertebrates, flies, and yeast, with some variations [15, 22–31].

Although the centromere protein functions are conserved among species especially in vertebrates, DNA sequences are more rapidly evolved and diverged [32, 33]. Human centromere chromatin currently locates on the repetitive DNA sequence, termed alpha-satellite DNA or alphoid DNA. The human centromeric alphoid DNAs have homogeneous tandem repeating structure composed of chromosome specific high order repeating (HOR) units, and each chromosome has different repeat length ranging from 0.5 to several Mb [34–36]. The centromeric chromatin (i.e. CENP-A-containing chromatin) is thought to occupy a portion of approximately several hundred kb of alphoid DNA, and H3K9me3-rich pericentromeric heterochromatin and CpG-methylated closed chromatin extends to the remaining region within the same repetitive DNA context [37–39]. H3K9me3 is a binding site for the HP1 protein, which promotes the cohesin assembly, ensuring bipolar attachment and equal segregation of sister chromatids [40–44]. Thus, both the centromere and heterochromatin are important for equal chromosome segregation.

The alphoid DNA has competence to form both centromere chromatin and heterochromatin in *de novo* mechanism [45–48]. Tens of kb of alphoid DNA input introduced into cells forms Mb-scale multimers that combine multiple introduced molecules. When the assembled chromatin distributions on the multimer are suitable for centromere function, the multimer is maintained as an extra chromosome independently from the host chromosomes. Such the extra chromosome is termed human artificial chromosome (HAC) [49, 50].

In addition to the centromere chromatin and heterochromatin, euchromatic modifications also exist on the alphoid DNA. Pioneering chromatin fiber microscopy revealed presence of euchromatic H3K4me2 modifications between CENP-A dense domains [37]. Immunoprecipitated CENP-A nucleosomes contain H4K20me1 modification, and removal of this modification abolished CENP-H and CENP-T assembly on centromere region [51]. Furthermore, forced heterochromatin intrusion to centromere chromatin inactivates centromere protein assembly and functions [47, 52–54]. In contrast to the heterochromatin spreading, a H3K14-acetyl transferase KAT7/HBO1/Mst2 prevents SUV39H1-mediated centromere inactivation [55, 56].

To date, numerous microscopic observations have revealed the existence of multiple histone modifications on centromere DNA sequence that regulate centromere chromatin assembly and function. On the other hand, non-microscopy techniques such as ChIP will also be important to clarify the distribution and regulatory mechanisms of these modifications around CENP-A containing regions in detail. It has been quite difficult to distinguish where the ChIP enriched fragments are located on the Mb-scale in homogeneous repetitive alphoid DNA context, but recent advances in deciphering the human genome sequences and the use of naturally occurring DNA mutations in the repeat sequences have enabled the mapping of CENP-A dense regions on the alphoid DNA [38, 39, 57]. However, in principal, ChIP mapping relies on natural mutations in homogeneous alphoid DNA repeats, limiting its location and resolution. For these reasons, detailed distribution of these histone modifications with CENP-A dense regions has not yet been analyzed.

In this study, to elucidate the centromere chromatin distribution in detail, we first created up to 192 kb of synthetic alphoid DNAs in which each repeat unit was combined with a unique and distinguishable short DNA barcode. Then, we generated HACs with the synthetic alphoid DNAs and performed ChIP-sequencing of the obtained HAC centromere with the DNA barcodes. As a result, two CENP-A dense regions larger than 18 kb were found per HAC, and the distribution of euchromatic histone modifications (H3K4me2, H3K14ac, H4K8ac, and H4K20me1) was positively correlated with the CENP-A dense regions. In contrast, distribution of heterochromatic histone modification (H3K9me3) and H3K36me2 was found to be negatively correlated with CENP-A dense regions (i.e. enriched in CENP-A sparse regions). These CENP-A dense and sparse regions inversely oscillated during the cell cycle. Whereas, the euchromatic modifications interdependently existed with CENP-A, and prevented the adjacent outer heterochromatin intrusion. These results provide insight into how and at what scale centromeric chromatin is maintained on the homogeneous alphoid DNA repeat contexts.

## Materials and methods

### Creation of synthetic repeat units and plasmid vectors

The 128 CenU units were generated by PCR using the common syn21F primer and primers specific to each CenU unit (CenU 001R to 128R) with the TW repeat DNA [63] as template. The 96 NCU units were also generated by PCR using the common syn21F primer and primers specific to each NCU unit (NCU 201R to 296R) with the Lm repeat DNA [63] as template. The PCR products for each unit contained a unique position code sequence, and these position code sequences are listed in Supplementary Table S1 and PCR primer sequences in Supplementary Table S2. The pNX31C plasmid DNA sequence was generated by an oligo DNA linker with pET30a vector DNA (69909, SIGMA) as a vector backbone. The sequence of this plasmid is shown in Supplementary Table S3. Each the repeating unit and vector backbone DNA above was digested with BamHI (1010A, TAKARA)-SpeI (R3133L, NEB) (odd number unit), NheI (R3131L, NEB)-NotI (R0189L, NEB) (even number unit), or BamHI-NotI (pNX31C vector DNA), respectively. These odd-numbered and even-numbered units and the pNX31C vector DNA fragment were applied to 0.8% Seaplaque GTG agarose (50110, LONZA) gel electrophoresis, and the DNA bands of interest were excised, extracted and ligated with T4 DNA ligase (2011A, TAKARA). The ligated DNA was transformed into DH5 alpha chemically competent cells (9057, TAKARA). Note that DNA ends cut by NheI and SpeI are cohesive and can be ligated, but ligation of NheI and SpeI ends results in no cleavage by either enzyme. This allows the same restriction enzyme and ligation combination to continue extending the doubled units in the designed order [45]. The pNX31C vector was used to clone inserts up to 16 ligated units. This vector was then replaced with bacterial artificial chromosome (BAC) vectors (pBAC KMN and pBAC Alone) to clone longer inserts. The pBAC Alone was generated by PCR using pBACNX plasmid DNA [45]. The pBACKMN contains a kanamycin/neomycin resistance gene cassette, whereas pBAC Alone lacks a selection marker for human cells. These plasmid DNA sequences are shown in Supplementary Table S3. BAC transformations to *Escherichia coli* cells were performed using a Gene Pulser Xcell instrument (Bio-Rad) into Electromax DH10B cells (18290015, Invitrogen) in 0.1 mm cuvettes at 1250 V/cm, 200 ohms, 25 µF.

### Cell culture

Human HT1080 and derived cells were cultured in the DMEM Glutamax I (10569010, Gibco) supplemented with the 10% tet-system approved fetal bovine serum (631 101, TAKARA) at 37°C and 5% CO_2_. The GENETICIN (11811–031, Gibco) was used at 400 µg/ml of active form for selection and maintenance.

### BAC DNA transfection to human cells

HT1080 cells were plated in a well of six-well plate. When the cells grown at approximately 80% confluency, BAC plasmids were transfected using 8 µl of ViaFect (E4981, Promega) diluted in 100 µl of the Opti-Mem I (31985062, gibco). After 24 h incubation, cells were trypsinized and transferred into three 10-cm dishes. Drug selection with 400 µg/ml of the active GENETICIN was started in the same time to the transfer. Untransfected cells were treated in the same way. After 2 weeks, we confirmed that all untransfected cells had died, and then harvested the transformed cells as bulk population. For single cell lines isolation, each colony was picked into a well of a 24-well plate. Each picked cell was cultured up to the scale of a six-well plate for genomic DNA analysis and a 10-cm dish for FISH. Cells were suspended in 1 ml of the Cellmenity cell stock solution (CM-3150, WakenBtech) and stored at −80°C.

### siRNA transfection

For siRNA transfection, 4 µl of Lipofectamine 2000 (11668027, ThermoFisher) and 100 µl of Opti-Mem I were used to transfect 200 pmol of siRNA into a well of a six-well plate at 80% confluence. The used siRNA sequences were siControl (AM4611, Ambion), KAT7 (s253, Ambion) [56], and HJURP [19]. The transfected cells were transferred to next six-well plate at an appropriate confluence and retransfected with the same amount of siRNA 72 h after the first transfection. Then after another 24 h, the cells were transferred into two 10-cm dishes. A total of 144 h after the initial transfection, cells were harvested for ChIP and RNA analysis. Immunoprecipitated DNAs or harvested total RNA was quantified by real-time PCR. The primer sequences for quantification are Shown in Supplementary Table S4. See also the ChIP section below.

### Metaphase FISH

To enrich for mitotic cells, 80% confluent cells in a 10-cm dish were treated with 20 ng/ml colcemid (15212-012, Gibco) for 5 h. Mitotic cells were collected by pipetting and washed once with PBS. Then, cells were suspended in a hypotonic cytospin buffer (10 mM Tris pH7.5, 0.2 mM EDTA, and 75 mM KCl) and incubated at room temperature for 5–10 min. During incubation, the slide (S024410, Matsunami), coverslip (C022221, Matsunami), filter card (M965FW, Simport), and Cytofunnel (M964-1, Simport) were sandwiched between a Cytoclip (M964B, Simport). In this case, mitotic cells were spread on a coverslip. Seventy microliters of cells incubated in the cytospin buffer were loaded onto a cytofunnel and centrifuged in a cytospin 4 (A78300003, Wakenyaku) at 2000 rpm for 5 min. The coverslips with chromosome spreads were stood on a rack and incubated in air at room temperature for 30 min, and then immersed and fixed in PBS (14249-24w, Nacalai) containing 2% formaldehyde (163-20145, Wako) for 5 min. Fixed samples were immersed in a 3:1 methanol/acetic acid solution for 15 min and then air-dried. Dried samples were immersed in PBS containing 0.1% Triton-X 100 (30525-89-4, Wako) (tPBS) for 30 min at room temperature, immersed in 2× SSC (32146–91, Nacalai) at 73°C for 3 min, and then immersed in 2× SSC containing 70% formamide (068-00 426, Wako) for 3 min to denature the DNA. After denaturation, chromosome samples were immersed in ice-cold 70% ethanol for 3 min, then immersed twice in 100% ethanol for 2 min at room temperature, and air-dried. For hybridization, the LacO-TAMRA PNA probe (TAMRA-oo-GTGAGCGGATAACAATT; custom, Panagene) was incubated in hybridization buffer (20 mM Na_2_HPO_4_, pH 7.4, 20 mM Tris, pH 7.4, 60% formamide, 2× SSC, and 0.1 μg/ml salmon sperm DNA) at 80°C for 5 min, quenched in ice-water for 3 min, and then hybridized with the chromosome samples on the coverslips overnight at 37°C in an air incubator. After hybridization, the samples were washed twice with washing solution (2× SSC containing 0.1% Tween-20) at 60°C for 10 min. The samples were rinsed with washing solution for 1 min at room temperature. Then, the samples were stained with 2× SSC containing 4’,6-diamidino-2-phenylindole (DAPI) (50 ng/ml) for 3 min, immersed in 2× SSC for 2 min, and the coverslips were mounted on slides using the VECTASHIELD Mounting Medium (H-1000, Vector) and a sealant for microscopic observation.

### FISH and immunofluorescence

To mount cell samples on coverslips, the 22 mm × 22 mm coverslips were placed in a six-well dish and cells were cultured on the coverslips for 2 days. For fixation, the medium was sucked out from the six-well dish, 1 ml of PBS containing 2% formalin was added, and the cells were incubated at room temperature for 10 min. Then, 2.5 M glycine was added to a final concentration of 125 mM, and the mixture was aspirated after 2 min, and 1 ml of the tPBS buffer was added to each well. For the antibody reaction, a primary antibody reaction buffer [tPBS containing 2% BSA (B6917, Sigma) and 0.1 ng/ml anti-human CENP-A antibody (A1, Masumoto lab)] was prepared, and 600 µl of the antibody reaction buffer was added to each well from which PBS had been sucked out. After incubation at 37°C for 60 min, the primary antibody reaction buffer was sucked out, and then secondary antibody reaction buffer [tPBS containing 2% BSA and 1/5000 diluted anti-mouse IgG conjugated Alexa 488 (A-11001, Invitrogen)] was added, followed by reaction at 37°C for 60 min. After secondary antibody reaction, cells were fixed with PBS containing 2% formalin and quenched with 125 mM of glycine, again. The fixed cells were then subjected to methanol-acetic acid treatment and denaturation, hybridization with the LacO-TAMRA PNA probe, and DAPI staining in the same manner as in the above section (Metaphase FISH), and then mounted on a slide glass.

### Image quantification

Cell images were acquired on an Axio Observer.Z1 (Zeiss) equipped with an LSM700 scan module and an Objective Plan-Apochromat 633/1.46 oil lens (Zeiss) using ZEN 2009 software (Zeiss). For image quantification, Z-stack images were acquired at 0.22 mm intervals. Maximum intensity projections of the resulting slices were created with ZEN software, and ImageJ 1.54j, (NIH) [58] or Fiji 1.0 [59] software was used for image quantification.

### ChIP

Approximately 2 × 10^7^ cells were harvested in a 50 ml tube by trypsinization, suspended in the culture medium, and centrifuged at 1200 rpm for 3 min in a swing rotor. The cells were thoroughly resuspended in 20 ml of PBS and fixed with 1/16 volume of 16% formalin solution (15700, EMS) at room temperature for 10 min. Formalin fixation was quenched by adding 1/20 volume of 2.5 M glycine and leaving at room temperature for 3 min. The fixed cells were centrifuged, the supernatant was sucked out, and the cells were suspended in 2 ml of 10T1E (10 mM Tris 8.0, 1 mM EDTA) and dispensed in 0.5 ml portions into 1.5 ml tubes. The dispensed cells were centrifuged at 6 000 rpm for 2 min, the supernatant was sucked out, and the cells were suspended in 100 µl of 10T1E containing 1/100 volume of the protease inhibitors cocktail (P8340, Sigma) (10T1E PIC). The resuspended cells were frozen and stored at −80°C until use in ChIP analysis. To begin ChIP, 350 µl of 10T1E PIC was added to frozen, fixed cell samples, and thawed on ice. Cells were sonicated in a Picoruptor 2 instrument (Diagenode) for 20 min at 4°C with 30 s on/off cycles. The sonicated cells were centrifuged at 15 000 rpm for 8 min, the supernatant was collected, and a portion of this supernatant was taken as the input sample. To the remaining cells, four volumes of the ChIP buffer (20 mM Tris 8.0, 600 mM NaCl, 1 mM EDTA, 0.1% SDS, 1.0% Triton-X100, 10% glycerol, and 1/1000 volume of protease inhibitor cocktail) was added, followed by addition of 10% BSA to a final concentration of 1%. These 1.5 ml tubes were centrifuged at 15 000 rpm for 8 min, and the supernatants were collected in new tubes and used for ChIP analysis. Twelve microliters of anti-mouse IgG Dynabeads (DB11201, ThermoFisher) were dispensed per sample, the beads were collected on a magnetic stand, and the supernatant was removed. The collected beads were washed once with ChIP buffer, suspended in 300 µl of ChIP buffer containing 1% BSA and 1 µg of mouse antibody, and rotated at 4°C for at least 60 min. Mouse antibodies used were: anti-CENP-A (A1), anti-CENP-B (5E6C1, Masumoto Lab), anti-H3K4me2 (300–34 803, MABI), anti-H3K14ac (7G8, Kimura Lab), anti-H4K8ac (382–09 091, MABI), anti-H4K20me1 (0421, MABI), anti-H3K36me2 (305–95 273, MABI), and anti-H3K9me3 (0308, MABI) [60]. The beads reacted with the antibody were collected using a magnetic stand, mixed with the ChIP sample, and rotated overnight at 4°C. The next day, the beads were collected on a magnetic stand, washed three times with 200 µl of ChIP buffer, and then suspended in 200 µl of 10T1E containing 2 mg/ml proteinase K (161-28 701, Wako) and incubated at 65°C for >4 h. To the incubated samples, 200 µl of phenol/chloroform/isoamyl alcohol mixture (25970-56, Nacalai) was added, mixed thoroughly using a vortex mixer, and incubated at room temperature for 3 min. The sample was mixed thoroughly again using a vortex mixer, then centrifuged at 10 000 rpm for 3 min, and the supernatant was collected in a separate tube. The ChIPed DNA was isopropanol precipitated, rinsed with 80% ethanol, and then suspended in 50 µl of 10T1E. Input DNA was also recovered in the same manner. The resuspended ChIPed DNA and a portion of the input DNA were used in real-time PCR to calculate the recovery rate. The primers used for real-time PCR are shown in Supplementary Table S4.

### Detection of the ratio of each PC DNA ratio by short-read sequencing

The PC DNA sequences contained in genomic DNA, ChIPed DNA, or reverse-transcribed cDNA were amplified in parallel by a competitive PCR using the common uniF and uniR primers, keeping the quantitative ratio of the original template DNA [45] (see also Supplementary Fig. S2A and Supplementary Table S4). The amplified PC sequences were indexed by PCR using the several primer sets (PCuni index F + 0∼3 and PCuni index R A1∼27 + 0∼3) that conferred indexes for use in Ilumina short-read sequencing. Note that each the primer sets contains an insertion of 0 to 3 bases after each index to ensure that the fluorescent colors of each sequencing spot are not the same when the common sequence region of the PC DNA is read. These primer sequences are shown in Supplementary Table S4. Each the indexed DNA was purified twice using the Ampure XP beads (A63880, Beckman). The size and amount of each DNA sample was then measured using bioanalyzer (Agilent), and the samples were mixed at appropriate concentrations to create pooled libraries for sequencing. The pooled libraries were applied to an Illumina MiSeq instrument and sequence reads were generated. The obtained sequence file was subjected to the cutadapt 4.0 with Python 3.9.13 [61] to remove unintended sequences. The PC DNA index list was processed by the bowtie-build (1.2.3) [62]. The list of PC DNA sequences is shown in Supplementary Table S1. The cutadapt-processed sequences were matched to the index list of PC DNA using the bowtie, and the number of reads for each PC was obtained using the samtools idxstats (1.11) [63]. The copy numbers of each PC DNA in genomic DNA were calculated based on the ratio of each PC DNA obtained by short-read sequencing and the quantification value of PC DNA by qPCR (see also Fig. 2A). The calculated values were almost consistent with the copy numbers obtained by long-read sequencing (Fig. 3A). For ChIP and cDNA analysis, the ratio of the number of reads obtained was assigned taking into account the copy number of each PC DNA obtained from the genomic DNA analysis, and the DNA recovery rate or RNA amount of each PC was calculated based on this assignment and the quantification value of PC DNA by qPCR.

### Long read sequencing of HAC centromere region

**Figure 1.**
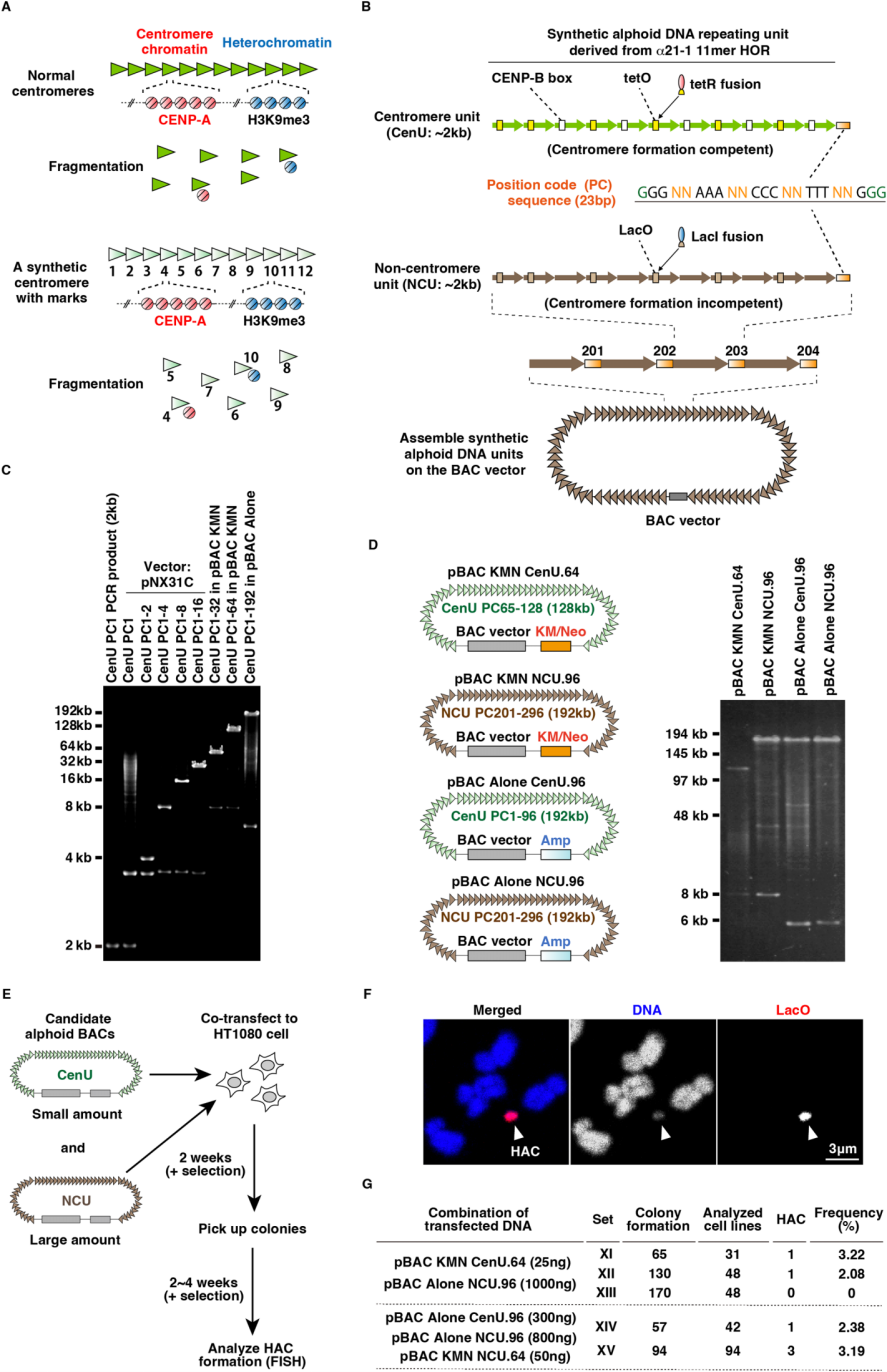
Generation of structural-analysis capable HAC. (**A**) Normal human centromeres are formed on highly repetitive sequence. Sheared repetitive DNA fragments are indistinguishable since they have the same sequence. If each repeating unit contained a specific identifying sequence, the cut DNA could be identified. (**B**) Created synthetic alphoid DNA repeating units. Alphoid DNA units were made from human alpha 21-I alphoid DNA High Order Repeat (HOR) unit. Each the centromere unit (CenU) contains 5 CENP-B boxes, binding site for CENP-B protein, 6 tetracycline operators (tetO), and a unique position code (PC) sequence. Each the non-centromere unit (NCU) contains 6 lactose operators (LacO), and a unique position code sequence. Since the CENP-B box sequences are required *de novo* centromere formation, the CenU has competence to form de novo centromere but the NCU not. These synthetic repeats are assembled one by one with restriction enzyme digestion and ligation procedure. (**C**) Pulse Field Gel Electrophoresis (PFGE). Each repeating unit was assembled by ligation-based plasmid cloning. A set of plasmid DNAs were digested with NheI and SpeI, and the resulting fragments were separated by PFGE. Insert size are indicated at the left side. (**D**) A set of plasmids used in this study. (Left) Combinations of human marker gene (Neo) and synthetic alphoid DNA (CenU or NCU) carried on a BAC plasmid. (Right) the set of plasmid DNAs were digested by NheI and SpeI, and separated by PFGE. (**E**) A schematic of HAC assay with cotransfection. (**F**) FISH example of an obtained HAC candidate clone (HAC 1–20). Mitotic chromosome spread was hybridized with a fluorescent conjugated probe for LacO sequence (red). DNA was stained with DAPI; bar: 3 µm. (**G**) Summary of the HAC assay. A total of 516 colonies were obtained, of which 263 cell lines that passed CenU DNA copy number screening by genomic PCR were subjected to FISH analysis, and finally six candidate HAC cell lines were obtained. The frequency in the rightmost column indicates the percentage of HAC containing cell lines among total analized cell lines. HAC-containing cell lines are defined as those in which >50% of the cell population contains HAC [45, 50].

To determine the DNA sequence of the HAC centromere region, genomic DNA was extracted using the GenomeTip100 (10243, QIAGEN). To efficiently enrich for HAC centromeric DNA sequences, the genome DNA was treated with a combination of the restriction enzymes AvrII (R0174L) and NsiI (R0127L), which do not cut CenU DNA. To enrich for uncut DNA, 300 µg of the digested genomic DNA was applied to a 0.8% SeqPlaque GTG agarose gel and separated in a Mupid mini gel electrophoresis apparatus at 100 V for 18 min. Note that before applying, the DNA sample was warmed at 60°C for 3 min. After electrophoresis, the agarose was excised from the origin of the well to 0.7 cm downstream to recover uncut DNA. The excised gel was then dissolved at 65°C for 7 min, and the agarose was digested using beta-agarase (M0392L, NEB) at 37°C for overnight. The amount of the beta-agarase used was 1U per 100 µl of gel volume. The beta-agarase-treated sample was cooled at 4°C for 15 min and centrifuged at 13 200 rpm for 15 min to collect the supernatant. DNA was recovered from the collected supernatant by isopropanol precipitation and suspended in 10T1E. The recovered DNA was purified using the AMPure PB beads (100-265-900, PacBio), combined into one tube, and shared on the Megaraptor 3 apparatus (Diagenode) at 300 µl with Speed 29. The shared DNA was collected using AMPure PB beads, and the DNA concentration was measured using the Qubit dsDNA BR kit (Q32850, ThermoFisher). The recovered DNA was subjected to removal of single strand overhangs, DNA damage repair, A-tailing, and addition of smart bell adapters using the SMARTbell Express Template Prep Kit 3.0 (102-182-700, PacBio). The Smart Bell library was purified with AMPure PB beads. The recovered library was subjected to agarose gel electrophoresis on Pippin HT (SageScience, Beverly, MA, USA), and DNA fragments >15 kb were harvested using the AMPure PB beads. DNA concentration was quantified using the Qubit dsDNA BR kit, and 300 pmol of SMRTbell DNA library was applied to a SMRT cell with standard sequencing primer (102-797-700, PacBio) and sequencing polymerase (102-797-300, PacBio). HiFi reads obtained by Revio (PacBio) were aligned on the sequences of the pBAC Alone CenU.96, pBAC KMN NCU.96, and pBAC Alone NCU.96 (Fig. 1D) as a reference by Minimap2 2.22 [64] to omit unnecessary genomic sequences and to select HiFi reads containing the desired HAC PC sequences. The selected HiFi read were assembled using hifiasm program (Version: 0.25.0-r726) [65]. The order and orientation of PC and BAC vector sequences on the assembled contig sequences were detected using the Fuzznuc 6.6.0 [66]. The resulting contig sequences are shown in Supplementary Contigs 1 (for HAC1-20) and Supplementary Contigs 2 (for HAC4-65).

### Quantification and statical analysis

Statistical test methods, numbers of independent experiments, and *P*-values are described in the figure legends. Statistical calculations were performed using Microsoft Excel functions. Significant differences in ChIP recovery rates, distribution of ChIP recovery in each CENP-A dense or sparse region, and CENP-A immunostaining intensity were tested using *t* tests. Data from multiple measurements are presented as mean ± SD.

## Results

### Generation of candidate HACs with a minimum centromere DNA

To generate structural analysis-capable HAC, we firstly created two types of synthetic HOR DNAs, a centromere forming unit (termed as CenU) and a non-centromere unit (termed as NCU), based on alpha 21-I 11mer HOR sequence derived from human chromosome 21 (Fig. 1A and B) [67]. The CenU or NCU sequences was designed to form the centromere or remaining regions of HAC, respectively. Each the CenU contains 5 CENP-B box sequences, the binding site of CENP-B protein [68]. These CENP-B box and protein are required for de novo centromere formation on the alphoid DNA in a density dependent manner [46, 47, 69–72]. The NCU sequence is nearly identical to the CenU, but each CENP-B box has two nucleotide substitution that prevents CENP-B binding and therefore cannot form centromeres [45]. Each the synthetic HOR unit was combined with additional unique 23 nucleotides sequence (termed position code: PC) for identification (Fig. 1B and Supplementary Table S1). Then, each the unit with the unique PC was tandemly assembled one by one to the 32 units (64 kb), using multiple ligation steps combined with plasmids cloning (Fig. 1B and C, and Supplementary Fig. S1A and B). To confirm competence and incompetence of CENP-A assembly, the 64 kb fragment CenU.32 or NCU.32 was separately transfected to human HT1080 cell, and bulk population of transformants were harvested after 2 weeks under geneticin selection and used for ChIP-qPCR (Supplementary Fig. S1C). In result, only the CenU DNA had enrichment with both anti-CENP-A and anti-CENP-B antibodies as expected (Supplementary Fig. S1C and D), indicating the CENP-B box dependent CENP-A chromatin assembly.

**Figure 2.**
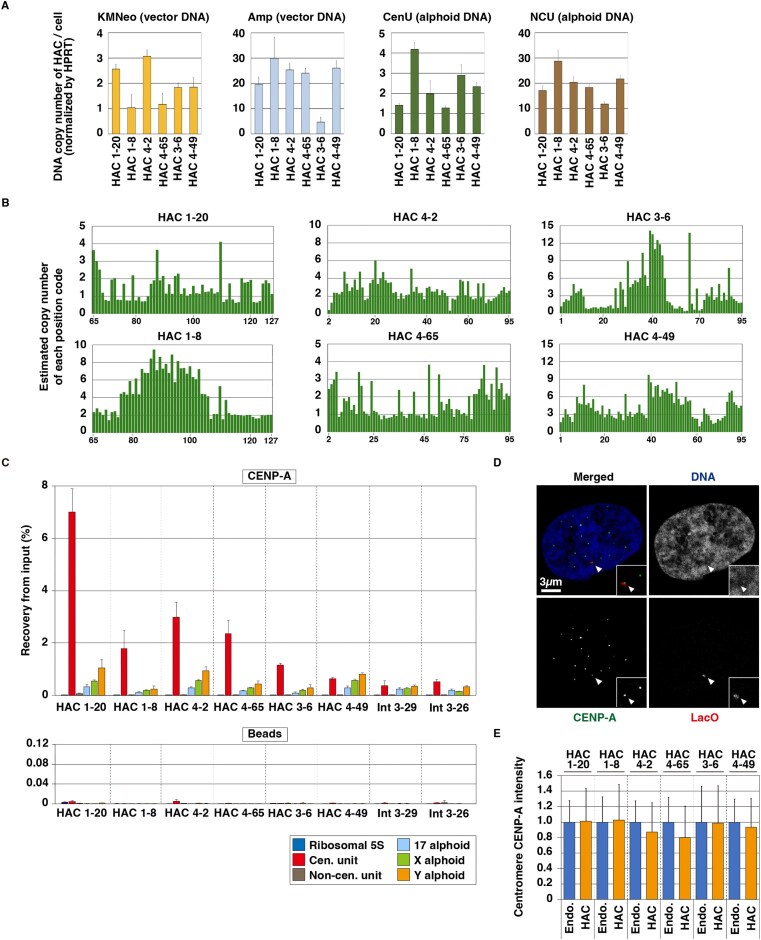
Synthetic HAC centromeres assembled on artificial repeat sequences. (**A**) Estimation of the copy numbers of CenU and NCU DNA per cell in HAC cell lines. Genomic DNA was extracted from HAC cell lines and quantified by qPCR (*n* = 3). Error bars, S.D. (**B**) Estimated copy number of each position codes. All CenU position codes were amplified in parallel by a competitive PCR, and the distribution ratio of each code was determined by short-read sequencing (see also Supplementary Fig. S2). The copy number of each code was estimated according to this ratio. (**C**) ChIP analysis. The obtained 6 HAC cell lines and 2 integrants (cell lines in which input DNA had integrated into the host chromosome) were analyzed by ChIP using CENP-A antibody or mock (beads only). Recovered DNA was quantified by qPCR (*n* = 3). (**D**) FISH and Immunostaining (an example of HAC 1–20 cell line). HAC was stained by FISH using LacO DNA probe and CENP-A protein was stained with anti-CENP-A antibody. DNA stained with DAPI (blue); Bar: 3 µm. Arrowheads indicate HAC location. (**E**) CENP-A intensity of HAC centromere. HAC CENP-A intensity (orange) and endogenous centromere (blue) were quantified (*n* > 16 cells). Endogenous centromere signal level was normalized as 1.

When elucidating the HAC centromere DNA structure, analysis becomes possible and easier if the copy number of CenU DNA is closer to one. For this reason, it is important to adjust the amount of introduced CenU DNA and avoid unnecessary multimerization as much as possible. We then estimated a minimum amount of the CenU DNA required for HAC centromere formation. By co-transfection of the CenU.32 and NCU.32 DNAs, HAC was generated as a heteromultimer of these input DNAs, and 20 ng of the CenU DNA per 3.5 cm dish transfection scale was sufficient for such the heteromultimer HAC formation (Supplementary Fig. S1E–G). In addition, the copy number of CenU.32 in each HAC cell line ranged from 6 to 236, with 7/19 of the HAC cell lines having a copy number <10 (Supplementary Fig. S1H). Therefore, it was conceivable that at most 320 copies of the CenU (∼640 kb) or less is sufficient for HAC centromere formation. We then considered increasing the unique copy number of CenU DNAs on the input BAC vector, and finally created tandem 64- or 96- mer repeat of unique CenU DNA with approximately 128 or 192 kb length (CenU.64 or CenU.96) (Fig. 1D). We also tried but could not obtained more longer ones, possibly due to the limitation of our BAC cloning system. We then decided to generate HAC using a minimum amount of the CenU.64 or CenU.96 by co-transfection with a sufficient amount of NCU.96 DNAs for remaining chromosomal domain formation (Fig. 1E). As results, total of six candidate HAC lines were obtained from FISH screening of 263 transformants, and the copy number of CenU.64 or 96 contained in each HAC cell line ranged from approximately 1–4 (Figs 1F and G, and 2A).

### CENP-A dense regions were formed on the synthetic centromere DNA sequence

To clarify copy number of the CenU PC DNA in the six candidate HAC cell lines, we then parallelly and quantitatively amplified the DNA region containing all the PC from genomic DNA by PCR with a single common primer set (Supplementary Fig. S2A) [45]. Such the parallelly amplified PCR products were subsequently applied to short read sequencing, and copy number of the CenU PCs were estimated with the sequencing results combined with the qPCR in Fig. 2A (Fig. 2A and B, and Supplementary Fig. S2B). As results, two candidate cell lines, the HAC 1–20 and HAC 4–65, were found to have less redundant CenU DNAs than the others, and contained more single-copy unique PCs (36 in the HAC 1–20 and 42 in the HAC 4–65) (Fig. 2B).

Next, we clarified whether centromere chromatin is formed on the CenU DNA sequence region of the obtained six candidate HAC by ChIP analysis of CENP-A. Significant recovery of CenU DNA, but not NCU, was detected in each candidate, and the recovery rates were 2–10 times higher than the endogenous X chromosome alphoid DNA (Fig. 2C). The CENP-A assembly level on the HAC was also confirmed by CENP-A immunostaining and quantification of its intensity. The CENP-A assembly amounts on each HAC was almost the same level to the endogenous centromeres (Fig. 2D and E). These results indicating that CENP-A dense HAC centromere core region was created in the relatively short CenU DNA region as expected, and that other long endogenous centromere DNA regions may also include the similar scale of CENP-A dense region, but there were many extra alphoid DNA copies where CENP-A is not assembled. These differences would be reflected in the ChIP recovery rate (Fig. 2C). We then analyzed CENP-A distribution on each the PC DNA region by the sequencing of ChIPed CenU PC DNAs, and found that in the two cell lines, HAC 1–20 and HAC 4–65, the CENP-A dense region mainly existed on the unique PC DNA rich region when compared to the other four HAC cell lines (Fig. 2B and Supplementary Fig. S2C). Such the feature had advantages for more detailed analysis of centromere chromatin structure.

Finally, we then cultured these HAC 1–20 and HAC 4–65 cell lines under non-selective drug conditions and analyzed HAC loss rate by FISH. The results showed that the HAC loss rates per cell division for HAC 1–20 and HAC 4–65 were 0.135% and 0.16%, respectively, indicating stability comparable to previously observed HACs [45, 50]. Based on these results, we choose the HAC 1–20 and HAC 4–65 cells for subsequent DNA structural analysis.

### Long-read sequencing revealed clustered HAC centromere structures

**Figure 3.**
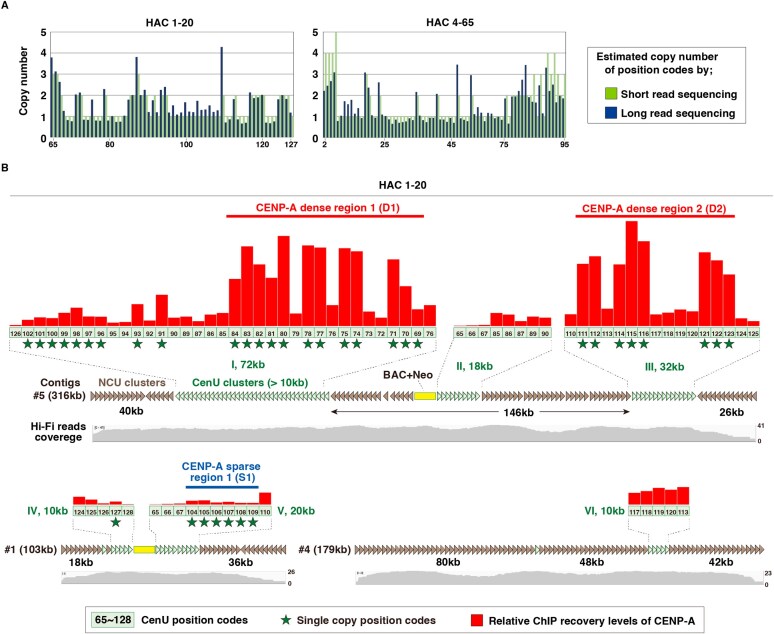
Decoding synthetic centromere DNA by the position codes and long-read sequencing. (**A**) CenU DNA copy number obtained with short read and long read sequencings. (**B**) Example of contigs containing CenU clusters obtained from long read sequencing of HAC 1–20 cell line. Clusters, which contained >5 CenU position code, are shown (I∼VI). The numbers in green boxes (65∼128) indicate the position codes of CenU DNA. Yellow boxes indicate BAC vector and KM/Neo marker gene cassettes. Green stars indicate that the position code above is a single copy. The gray histograms show the coverage of HiFi reads. Red bars, relative CENP-A ChIP recovery level based on Supplementary Fig. S2C. CENP-A dense regions are shown as D1 and D2. CENP-A sparse region is shown as S1.

To clarify how the HAC centromere region is organized, we performed long read DNA sequencing for the CenU DNA containing region. In HAC cell lines analyzed using long reads, copy number estimates from long and short reads in CenU PC were nearly concordant (Fig. 3A), indicating most of the CenU PCs were detected both short and long read sequencing. We then assembled obtained long read sequences using hifiasm. A total of 14 contigs (total length: 1.4 Mb) were obtained from HAC 1–20 cells. Three of these contigs contained six major clusters of continuous CenU DNA longer than 10 kb (Fig. 3B). Furthermore, throughout the contigs, junction sites were found where the introduced CenU DNA and NCU DNA molecules were linked in either the forward or reverse direction. Specifically, these repeat sequences were linked in the forward direction at 116 sites (94.3%) and in the reverse direction at 7 sites (5.7%). These results suggested a previously unknown mechanism for HAC multimerization in which, during the HAC formation, the cotransfected alphoid DNAs were primarily joined by homologous recombination and, in some remaining cases, may be joined by a nonhomologous end-joining mechanism. From the HAC4-65 cells, 17 contigs totaling 2.3 Mb were obtained, and in five of these contigs, seven major clusters of continuous CenU DNA longer than 10 kb were detected. In addition, 256 forward junctions (93.4%) and 18 reverse junctions (6.6%) were found between CenU DNA and NCU DNA (Supplementary Fig. S3).

Next, to clarify in which CenU clusters CENP-A is distributed, we assigned the CENP-A enrichment obtained in Fig. 2E to the PCs of each cluster. In the HAC 1–20 cells, CENP-A dense (high enrichment) regions (D1, 32 kb and D2, 26 kb) were present in the clusters I and III, and in the remaining clusters, there were CENP-A sparse (no or few enrichment) region (S1, 12 kb) in the cluster V (Fig. 3B). Comparing the unique PCs (indicated by green stars in Fig. 3B) in the CENP-A dense and sparse regions, there was a significant difference in the enrichment level of CENP-A (*P* < 0.001). Similarly, in the HAC 4–65 cells, CENP-A dense regions were present in the cluster I and II (D3, 50 kb and D4, 18 kb), and the sparse region (S2, 14 kb and S3, 4 kb) were present in the cluster VI and VII (*P* < 0.001) (Supplementary Fig. S3). In addition, CENP-A dense regions were found in the clusters which contained >18 kb of continuous CenU DNA region in the both cell lines. In our previous results, HAC and *de novo* centromere formation occurred when continuous 30 kb of alphoid DNA was used as an input, but not with 10 kb [72]. Considering with these results, a lower limit of continuous centromere formation competent DNA length required for de novo centromere chromatin formation and its epigenetic maintenance may be around 18 kb.

### CENP-A dense regions were enriched in euchromatic modifications

**Figure 4.**
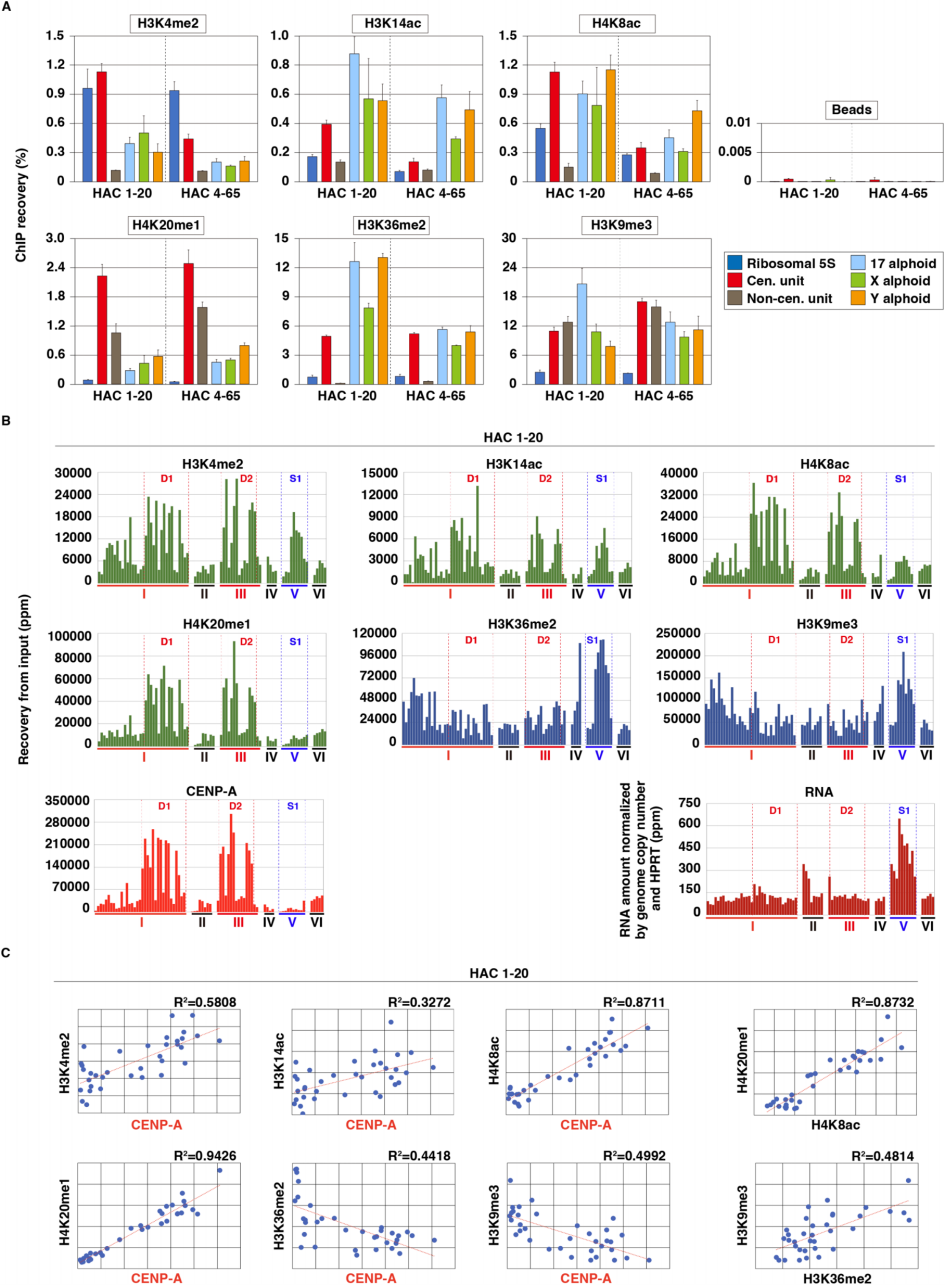
Histone modifications positively and negatively correlated with CENP-A dense regions. (**A**) ChIP-qPCR analysis of HAC cell lines. ChIP assay was carried out using normal asynchronous cells with indicated antibodies and beads alone. Recovered DNA was quantified by qPCR. Error bar, SD (*n* = 3). (**B**) Estimated ChIP recovery of each CenU position codes of the HAC 1–20 cell line. CenU position codes in each ChIPed DNA were sequenced and used the results to estimate recovery for each code (see also Supplementary Fig. S2). CENP-A dense (D1, D2) and sparse (S1) regions are shown in the graph. (**C**) Correlation plot of each position codes. Pearson’s correlation coefficients between CENP-A and each histone modification distribution among the position codes (R2) are displayed on the right shoulder of each graph.

As mentioned above, several specific histone modifications, H3K4me2, H3K14ac, H4K20me1, and H3K9me3, reported to exist around CENP-A containing chromatin region. However, most of these results were based on fluorescence microscopy, and needed to be verified in more detail using other methodologies. In addition, KAT7 introduces acetylation not only H3K14ac but also on histone H4, including H4K8ac [73–75], and H3K36me2 also exists on the alphoid DNA and assists CENP-A assembly [48, 76]. Then, we firstly confirmed whether these modifications are actually enriched in the CenU DNA regions by ChIP-qPCR assay in the HAC 1–20 and HAC 4–65 cells. In results, all these modifications were enriched at significant levels (Fig. 4A).

To investigate a more detailed distribution of these modifications on the CenU DNA, we next sequenced the ChIP-enriched CenU PC DNAs in the HAC 1–20 cells. In results, whole distribution of the histone H4 modifications, H4K8ac and H4K20me1, showed a significant positive correlation with CENP-A (*R* = 0.933 for H4K8ac, and 0.971 for H4K20me1), and these modifications were specifically high in the CENP-A dense region (D1 and D2) when compared to the sparse region (S1) (*P* < 0.001) (Fig. 4B and C). In addition, the distribution of H3K4me2 and H3K14ac modifications also showed positive correlation with CENP-A, although, these modifications were not specific at CENP-A dense region and were also distributed in other CenU DNA region. In contrast, the whole distribution of H3K9me3 and H3K36me2 showed negative correlation with CENP-A, and these modifications were high in the CENP-A sparse region when compared with dense region (*P* < 0.001). The same whole positive and negative correlation patterns were also observed in the HAC4-65 cells with significant level, and H4K8ac and H4K20me1 modifications were also high in the CENP-A dense region (D3 and D4) when compared to the sparse region (S2 and S3) (*P* < 0.001) (Supplementary Fig. S4A and B). However, in the HAC 4–65 cells, distribution of H3K36me2 and H3K9me3 were not specifically high in the CENP-A sparse region when compared with the dense region, and these modifications were also distributed in other CenU DNA region. Summarizing the results, H4K8ac and H4K20me1 may specifically coexist within the CENP-A dense region in the both cells. The other modifications, K3K4me2 and H3K14ac, were relatively high at the CENP-A dense regions but also exist on the CENP-A sparse regions. Invertedly, H3K36me2 and H3K9me3 were relatively high at the CENP-A sparse regions but also exist on the CENP-A dense regions at lower level. In other words, CENP-A chromatin coexisted with euchromatic modifications but showed a negative correlation with H3K36me2 or heterochromatic H3K9me3.

In addition, to determine whether the transcribed region of CenU DNA showed any correlation with CENP-A distribution, we harvested and analyzed total RNA using qPCR and the PC-sequencing. In the HAC 1–20 cells, CenU RNA was detected, but the RNA to DNA copy ratio was very low compared to the HPRT gene. With regard to transcriptional bias, the CENP-A sparse region (S1) had higher RNA enrichment compared to the dense regions (*P* < 0.001) (Fig. 4B). However, in the HAC 4–65 cells, CenU RNA levels were lower than in HAC 1–20, and no correlation was detected between the transcribed and CENP-A sparse regions (Fig. 4B and Supplementary Fig. S4B). At present, it is possible that the CenU DNA transcription level and region are regulated by factors other than CENP-A density.

### CENP-A and histone modification levels were fluctuated during the cell cycle

**Figure 5.**
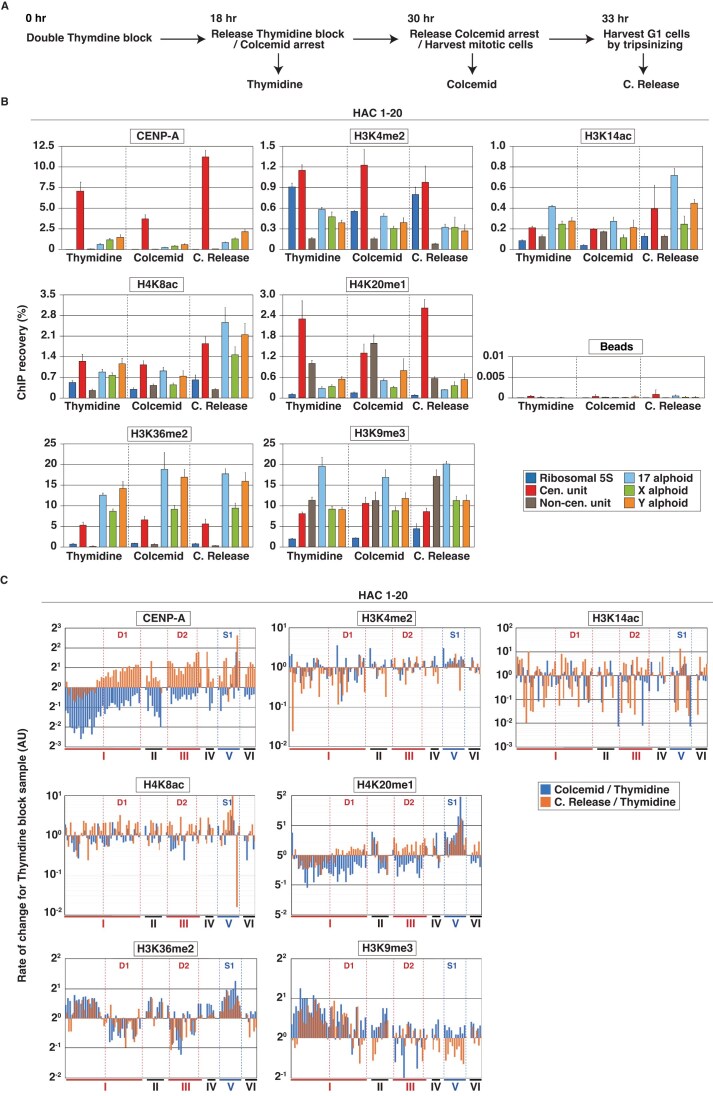
CENP-A assembly and histone modification levels oscillated during the cell cycle. (**A**) Time course of cell cycle synchronized sample collection. (**B**) ChIP-qPCR analysis of the HAC 1–20 cell lines. ChIP assay was carried out using the synchronized sample sets shown above with indicated antibodies and beads alone. Recovered DNA was quantified by qPCR. Error bar, SD (*n* = 3). (**C**) Rate of change in ChIP recovery relative to thymidine treated G1/S samples. CenU position codes in each ChIPed DNA were sequenced and used the results to estimate recovery for each code (see also Supplementary Fig. S2). Values from each sample were normalized to the value from the thymidine-treated sample. CENP-A dense (D1, D2) and sparse (S1) regions are shown in the graph.

Since the timings of CENP-A replenishment and DNA replication are different, CENP-A assembly and chromatin modifications levels around CENP-A dense region are expected to fluctuate during the cell cycle. We then compared three samples in different cell cycles by ChIP using HAC 1–20 cells (Fig. 5A). In results, compared with thymidine-blocked G1/S cells, the CENP-A assembly level decreased by almost half in the colcemid-arrested metaphase cells, and again increased in the colcemid-released G1 cells (Fig. 5A and B). The decrement in metaphase was consistent with previous cytological observations. Across all the three samples, short read sequence analysis of the CenU PCs confirmed significant changes in the CENP-A assembly level in the entire CENP-A dense regions (Thymdine-Colcemid, Colcemid-Colcemid Release, and Colcemid Release-Thymidine, D1 and D2, *P* < 0.001) (Fig. 5C). Similar changes were detected in HAC 4–65 cells (Thymdine-Colcemid and Colcemid-Colcemid Release, D3 and D4, *P* < 0.001), with the exception that no differences were observed between the colcemid-released and thymidine-blocked cells in the D4 region (*P *= 0.0647) (Supplementary Fig. S5A and B). These results indicated a CENP-A assembly cycle in which CENP-A density diluted during DNA replication, replenished in slight excess during the next G1 phase, and returns to its original level by the time it enters the next S phase (Fig. 5B and C).

Histone modifications during the cell cycle in CENP-A dense regions have not yet been investigated. We then investigated changes in the histone modifications commonly detected in HAC1-20 and HAC4-65 cells in the CENP-A dense regions (Fig. 5B and C). In result, there were common significant changes in the H4K20me1 and H3K9me3 level in all the three samples. In CENP-A dense regions, colcemid-arrested metaphase cells had less H4K20me1 compared with the thymidine-blocked G1S cells (D1, D2, D3, and D4, *P* < 0.001) or colcemid-released G1 cells, except in the D4 region (D1 and D2, *P* < 0.001; D3, *P* < 0.01; D4, *P* = 0.0169). These changes were similar to the case of CENP-A. There may be some relationship between the CENP-A deposition and H4K20me1 modification for further CCAN proteins assembly mechanism [51]. Changes in H3K9me3 levels in the CENP-A dense regions increased and decreased inversely to those of CENP-A. In the CENP-A dense region in the longer CenU DNA cluster (>60 kb), H3K9me3 increased in the colcemid-arrested metaphase cells (D1, *P* < 0.01; D3, *P* < 0.001), and in the shorter cluster (<30 kb), H3K9me3 decreased or remained unchanged (D3, *P* < 0.01; D4, *P *= 0.318). CENP-B box containing sequences, such as CenU DNA, are not only competent for de novo centromere formation, but also permissive for heterochromatinization to occur via CENP-B [46]. In the contiguous CENP-B-positive chromatin regions longer than the minimum length of CENP-A dense region, there may be room for an oscillation in which a decrease in CENP-A leads to an increase in H3K9me3 modification, or vice versa. On the other hand, since the shorter clusters were less intruded by H3K9me3, it is possible that there is some specific structure in the minimum CENP-A dense regions that are less susceptible to H3K9me3 intrusion, such as a CCAN assembly.

### Interdependent maintenance of CENP-A and coexisting modifications prevented heterochromatin spreading

**Figure 6.**
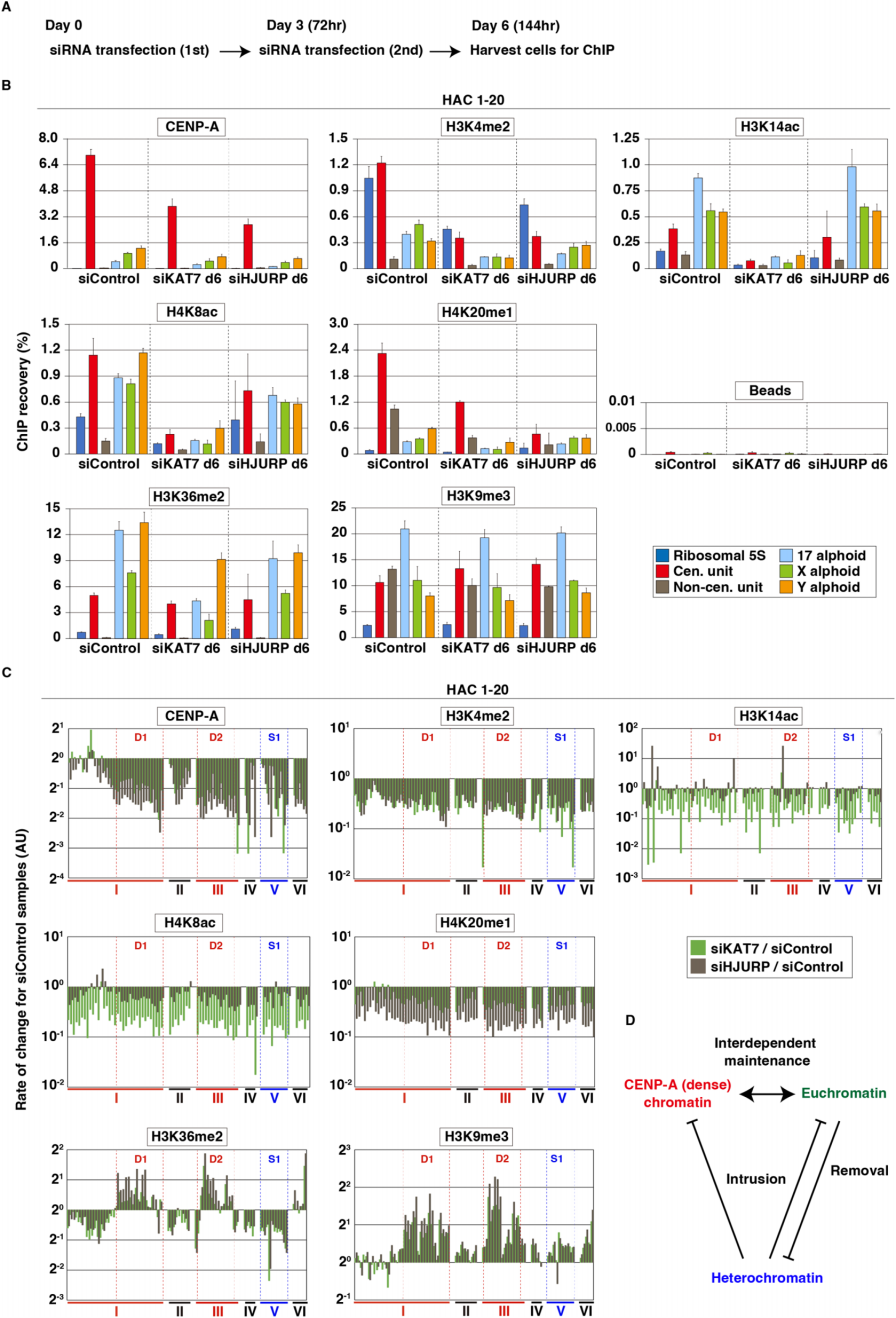
Centromere chromatin and euchromatin interdependently prevented heterochromatin intrusion. (**A**) Time course of KAT7 or HJURP knockdown experiment with siRNA. The siRNA was transfected twice (at day 0 and 3) for each sample, including controls (siControl). Harvested cells were used for ChIP analysis. (**B**) ChIP-qPCR analysis using mRNA knockdown in the HAC 1–20 cells. ChIP assay was carried out using the siRNA transfected cells shown in above with indicated antibodies and beads alone. Recovered DNA was quantified by qPCR. Error bar, SD (*n* = 3). (**C**) Rate of change in ChIP recovery relative to the siControl sample. CenU position codes in each ChIPed DNA were sequenced and used the results to estimate recovery for each code (See also Supplementary Fig. S2). Values from each sample were normalized to the value from the siControl sample. CENP-A dense (D1, D2) and sparse (S1) regions are shown in the graph. (**D**) The three-way relationship in the CENP-A dense region.

Forced introduction of H3K9me3 modification inactivates centromere function [47, 53, 54]. In this study, even during the normal cell cycle, an H3K9me3 and CENP-A oscillations occur (Fig. 5). To investigate how the histone modifications, which have a positive correlation with CENP-A, are involved in the oscillation of CENP-A and H3K9me3, we performed ChIP-sequence analysis under conditions where the acetyltransferase KAT7 or CENP-A deposition factor HJURP were depleted. In results, entire CENP-A assembly levels were decreased both HAC1-20 and HAC4-65 cells by either KAT7 or HJURP depletion (Fig. 6A and B, and Supplementary Fig. S6A and B). These results were consistent with previous reports [18, 19, 56]. In the CENP-A dense regions, changes were observed in almost all the analyzed histone modification levels, except H3K14ac and H3K36me2 (Fig. 6B and C, and Supplementary Fig. S6A and B). The analyzed modifications that were positively correlated with the CENP-A distribution decreased in the CENP-A-dense region, whereas the modification that were negatively correlated (i.e. H3K9me3 or H3K36me2) were increased under the absence of KAT7 or HJURP (D1, D2, D3, and D4, *P* < 0.001) (Fig. 6B and C, and Supplementary Fig. S6BC). This result suggests that KAT7 and HJURP work interdependently to maintain CENP-A assembly and histone modifications in the CENP-A dense region, and at the same time, these factors prevent H3K9me3 or H3K36me2 modifications from intrusion on the CENP-A dense regions.

The exceptions were observed in the H3K14ac levels under HJURP knockdown conditions and in the H3K14ac levels in the D2 region under KAT7 knockdown conditions, and no significant decrease in the amount of modification was observed in these regions (Fig. 6C and Supplementary Fig. S6C). The results suggesting that H3K14ac levels are mainly dependent on KAT7 and may not be on HJURP. In addition, when compared with the results between KAT7 and HJURP depletion, H4K20me1 reduction was more dependent on HJURP (D1, D2, and D3, *P* < 0.001; D4 = 0.011), and H4K8ac reduction was more dependent on KAT7, (D1, D2, D3, and D4, *P* < 0.001) respectively. These CENP-A replenish and histone acetylation pathways may end up prevent dilution of CENP-A density, thereby protecting the intrusion of CENP-A sparse domain-type histone modifications.

## Discussion

HACs have been utilized to elucidate requirements of DNA sequences and chromatin modifications for formation and maintenance of *de novo* centromeres [35, 77, 78]. Whereas, the highly homogeneous repetitive sequences used in HAC formation and the multimerization of input DNA making it extremely difficult to understand the detailed HAC DNA and chromatin structure. Here, we created HAC centromeres from synthetic satellite DNA with one PC sequence embedded every 2 kb repeating unit, and obtained structural information of the HAC centromere DNA and a high-resolution chromatin distribution.

Regarding centromeric chromatin formation, we found that CENP-A dense regions were formed on continuous centromere formation competent DNA sequences with a minimum size of approximately 18–50 kb (Fig. 3). This information will also be useful for the construction of the next synthetic centromere DNA. Furthermore, CENP-A dense regions were detected at two centromere DNA clusters in each of the two HAC cell lines. In one of these lines (HAC 1–20), the distance between these clusters was 146 kb. Although similar results have been reported for natural human centromeres [38], it was unclear why there are two regions instead of one. Very recently, high-resolution microscopy has demonstrated that two CENP-A dense spots exist on single chromosome and suggested the two-domains structure may have some functional advantages to the bipolar chromosome alignment [79]. There is possibility that *de novo* assembly of CENP-A at two sites is required to acquire full HAC centromere function, and the DNA distance between the two CENP-A dense regions may also be important for chromosome segregation. To verify this, a technology to synthesize more longer synthetic centromeric DNA as designed will be important.

Regarding HAC DNA structure, copy number analysis using the PC DNA did not detect any significant changes in the once-established HAC DNA structure. However, compared with the input BAC plasmid DNA, the sequences of the HAC DNA were rearranged at a frequency ranging from once every 10 kb to several tens of kb. With this rearrangement, multiple input DNA molecules containing CenU and NCU sequences formed large HAC molecules of Mb scale. These input DNA multimerization and rearrangements probably occurred at an early stage in HAC formation. During HAC formation, if HAC precursors are unable to establish functional centromeres or the DNA size is insufficient for stable HAC maintenance, the HAC precursors are excluded from chromosome segregation mechanism, form micronuclei, and subsequently undergo extensive DNA rearrangements via a chromothripsis mechanism [47, 80, 81]. Following this rearrangement, HAC precursors may eventually acquire both a minimal size for stable maintenance (approximately 750 kb in human cells) [82, 83] and functional centromere chromatin, and then re-enter the canonical nucleus after correct chromosome segregation as a HAC. Thereafter, the HAC appears to be stably maintained without further structural changes. With regard to this hypothesis, a very recent study demonstrated that HACs could be formed without a multimerization process by introducing a 750 kb YAC into cultured human cells and simultaneously establishing a human neocentromere by HJURP tethering to a flanking LacO repeat sequence [84, 85]. Although the complete DNA structure of this YAC-based HAC is not yet known, in the near future, it may be possible to create HACs with totally defined DNA structures. For example, if a single-copy HAC can be generated by combining the barcoded synthetic centromere repeat DNA of sufficient length and structure with an appropriate staff DNA sequence, it will be suitable for analyzing the entire HAC structure. This would reveal the positional relationships of chromatin regions such as centromeres, heterochromatin, inner centromeres, and replication origins on the DNA sequence, leading to a deeper understanding of the principles of chromosome maintenance.

### Chromatin modifications that regulate centromere structure

In this study, there were CENP-A dense and sparse regions, each of which corelated with different histone modifications. H3K9me3 modification, which inactivates centromeres, was negatively correlated with CENP-A distribution and found to be located almost adjacent to the CENP-A dense regions. During the cell cycle, H3K9me3 oscillated intrusion and eviction to CENP-A-dense regions, but was less evicted under conditions depleted of the KAT7 or HJURP. This result is consistent with previous [56]. KAT7 was also involved in the level of H4K8ac modification. CENP-A distribution was well correlated with H4K8ac, but not with the H3K14ac. Although H3K14ac promotes eviction/turnover of canonical H3 nucleosomes [86, 87], but not CENP-A, therefore H3K14ac containing nucleosome may be disappeared by eviction and CENP-A nucleosome may retain H4K8ac.

H4K20me1 was highly correlated with the CENP-A dense region. This correlation is consistent with the results in chicken cells [51]. The H4K20me1 modification is more likely to occur on CENP-A nucleosomes than on canonical H3 nucleosomes *in vitro* [88], and is more prominent after than before CENP-A deposition in cells [89]. Since H4K20me1 was more reduced by HJURP depletion than KAT7 (Fig. 6), there may be an H4K20me1 modification mechanism that functions in concert with the HJURP-mediated CENP-A deposition.

H3K4me2 is involved in the assembly of HJURP [76]. H3K4 di-methylases, MLL family proteins are localize to the centromere [90], and tethering of MLL to alphoid DNA increases CENP-A assembly amounts [87]. Although H3K4me2 was not fully correlated with CENP-A dense regions, it may promote HJURP assembly through a combination of several circumstances. Additionally, H3K36me2 is introduced by ASH1L in a CENP-B-dependent manner and is somehow involved in centromeric chromatin formation [48], but its distribution showed a negative correlation with CENP-A dense regions. It is interesting how H3K36me2 is positively involved in CENP-A assembly. Perhaps there is a preference for CENP-A deposition, which is more likely to occur on H3K36me2 regions than on H3K9me3-modified regions.

Overall, the CENP-A dense region contained many euchromatic modifications, while the sparse region immediately outside of it contained heterochromatic H3K9me3 modifications and H3K36me2. In addition to those investigated so far, it is possible that many more histone modifications and proteins are specifically assembled in the dense and sparse regions of CENP-A. The structurally analyze capable synthetic centromere sequences and HACs we had created are expected to be useful tools for elucidating the distribution and regulatory mechanisms of such unexplored centromere chromatin.

### The role of transcription in the centromere

Previous studies have detected euchromatin modifications, RNA polymerase II localization, and transcripts in the centromere, suggesting a correlation between these factors and centromere function and stable CENP-A deposition [91–93]. On the other hand, while centromere transcription itself contributes to CENP-A stabilization and chromatin opening, high levels of transcription are not necessarily present in regions with high CENP-A density [93, 94]. In this study, despite the detection of euchromatic modifications in the CENP-A dense regions, the transcription levels were low. These results suggest that even low-frequency transcription, close to the detection limit, may be sufficient for stable CENP-A deposition and function. To understand the role of centromere transcription, not only the amount of transcription but also the transcription process itself may be more important.

### Centromere structure analysis and applications of HAC

HAC can be transferred to other species cells using the Microcell Mediated Chromosome Transfer (MMCT) techniques [95–99]. This method may be suitable for comparative analysis of centromeric chromatin structure among species [93]. HACs may be useful for elucidating similarities and differences between species in chromosome functions, including centromeres. Furthermore, HACs are also useful as vectors [95, 98, 100] and are expected to be applied to the production of useful substances, control of cell functions, and medicine in a variety of organisms. Although the detailed structure of HAC had remained a black box, several recent advances have provided clues to understand its structure, which may lead to the control and application of HAC function in the near future.

## Supplementary Material

gkag597_Supplemental_Files

## Data Availability

The short-read sequence data used to count the position codes have been deposited in NCBI’s Gene Expression Omnibus (GEO) [101]. The GEO accession numbers for these data are GSE287040 (DNA), GSE287041 (RNA) and GSE287042 (ChIP). The contig sequences assembled from HAC long-read sequences are shown in the Supplementary data (Supplementary Contigs 1 and Supplementary Contigs 2). The raw sequence data used for this assembly is available upon request.
