# Supplementary material for: Epigenetic landscape of synthetic human centromere core regions: CENP-A assembly and euchromatic modifications interdependently antagonize heterochromatin accumulation
Source: Nucleic Acids Res. 2026 Jun 30;54(12):gkag597. doi: 10.1093/nar/gkag597 (PMC13314379; doi:10.1093/nar/gkag597)
Supplement: gkag597_Supplemental_Files [file gkag597_supplemental_files.zip › Supplementary Figures.pdf]

Supplemental Figures and Legends

Figure S1

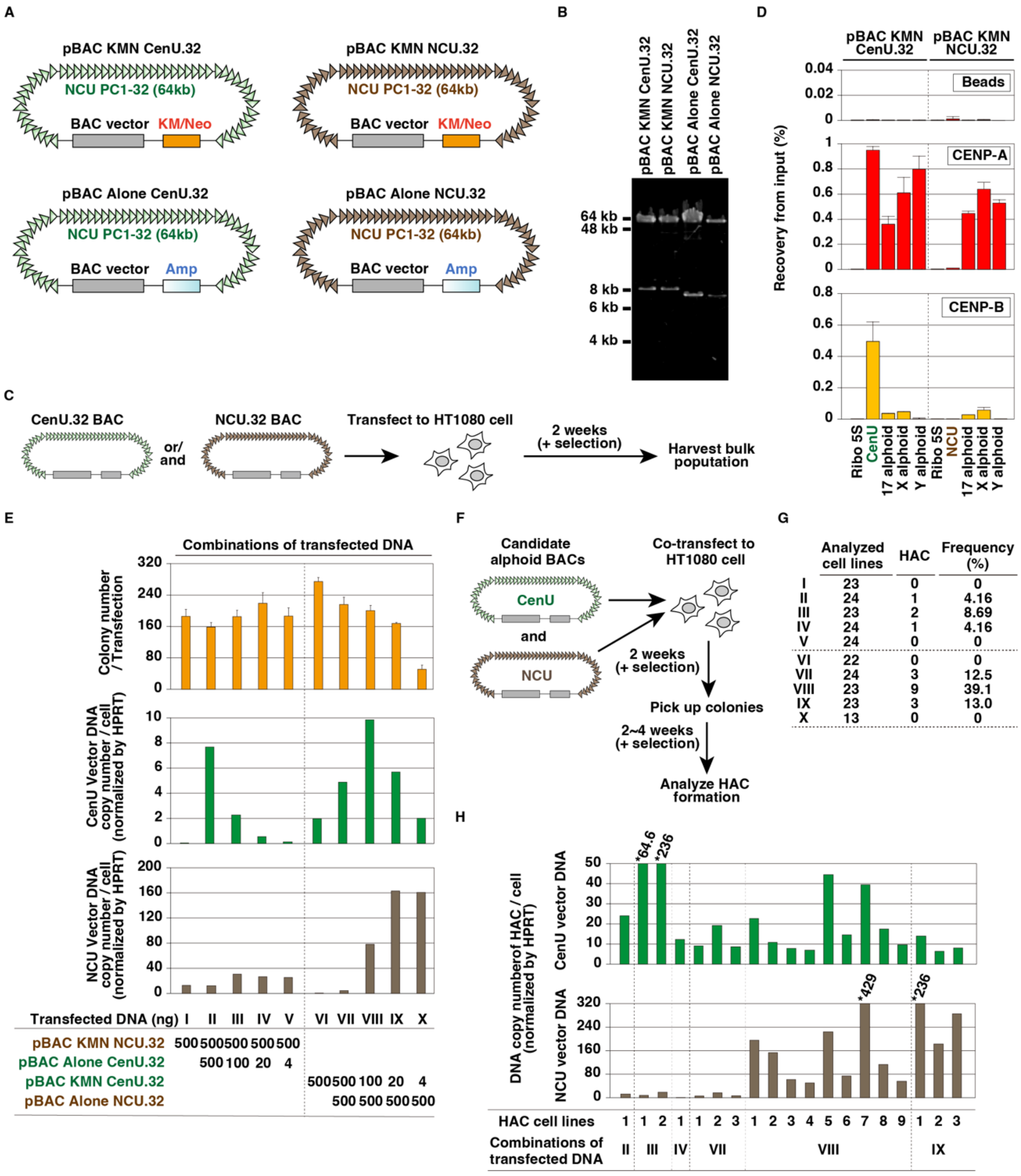

Supplementary Figure S1: Supplemental information for Figure 1

(**A**) A set of plasmids used in transient transfection assays. (**B**) PFGE of the plasmids shown in A. Plasmids were digested with NheI and SpeI, and the resulting fragments were separated by PFGE. (**C**) Scheme for transient transfection and ChIP analysis. (**D**) ChIP analysis. ChIP DNA recovery was quantified with qPCR. Error bars, SD (n=3). (**E**) Copy number estimation of the

CenU and NCU vector DNA introduced per cell. The CenU and NCU DNA were cotransfected into cells at different ratios. The cotransfected cells were cultured under drug selection (G418) for 2 weeks and colony numbers were counted. All the cells were then harvested together and genomic DNA was used for analysis as the bulk population. The copy number was estimated by qPCR. (**F**) A schematic of HAC assay with cotransfection. HAC formation was analyzed by FISH of metaphase chromosome spreads. (**G**) Summary of the HAC assay. The same amounts of DNA sets as in panel E were cotransfected. The frequency in the rightmost column indicates the percentage of HAC containing cell lines among total analized cell lines. HAC-containing cell lines are defined as those in which more than 50% of the cell population contains HAC (45,50). (**H**) Copy number estimation of the CenU and NCU vector DNA introduced per cell. HAC cell lines were cultured and genomic DNA were harvested and quantified by PCR.

Figure S2

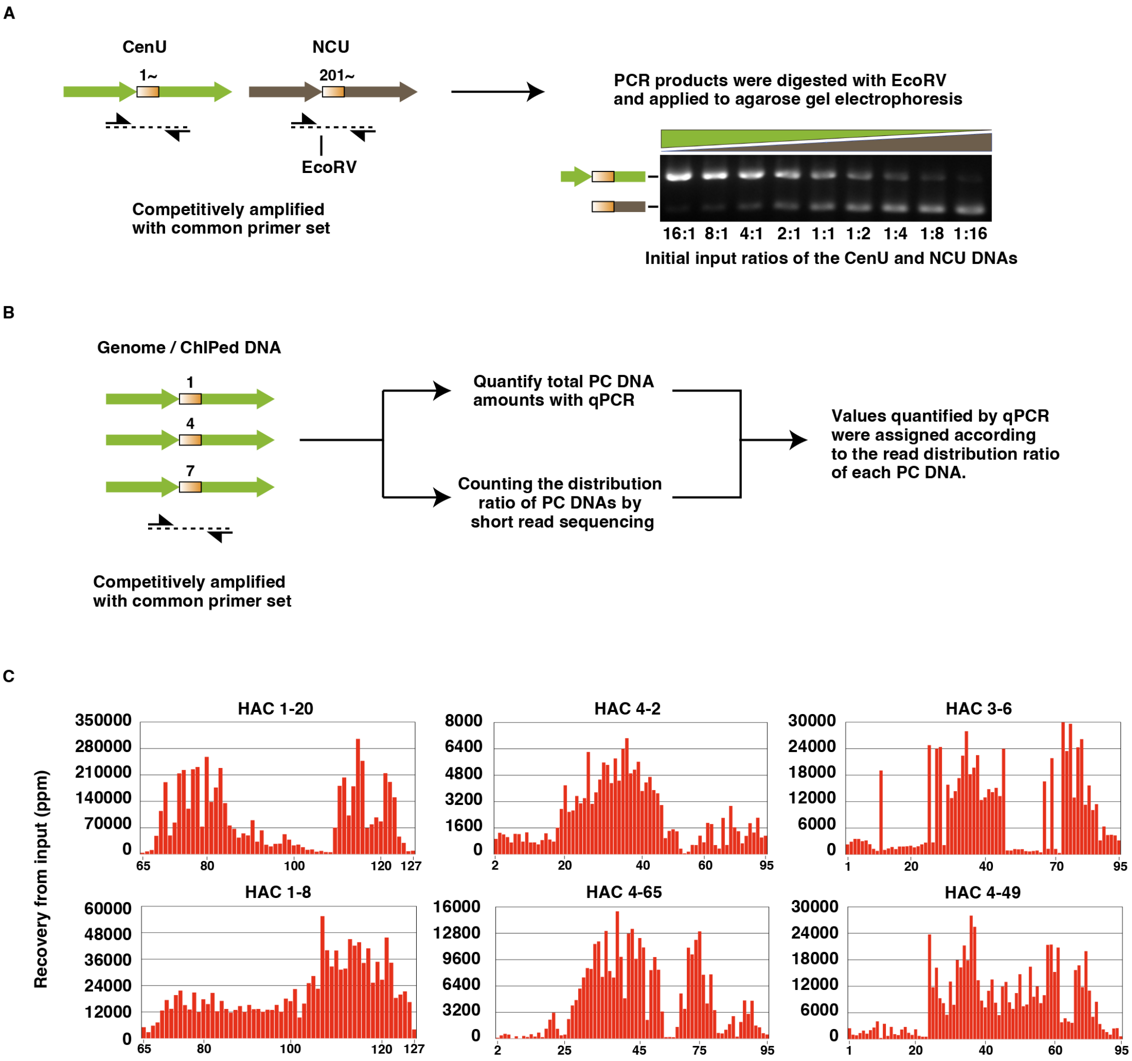

Supplementary Figure S2: Supplemental information for Figure 2

(**A**) Competitive PCR. (Left) The CenU and NCU position code sequences can be amplified using a common primer set, which allows amplification of the input DNA while keeping the initial PC DNA ratio. (Right) An image in which DNA fragments amplified by competitive PCR were digested with EcoRV and then separated by agarose gel electrophoresis. The CenU-derived fragments not containing the EcoRV site (upper bands) and NCU-derived fragments containing a EcoRV site (lower bands) were separated, and these were amplified while maintaining the quantitative ratio of the initial input DNA. (**B**) Estimation of the proportion of position code DNA in PCR products. Genomic DNA, ChIPed DNA, or RNA was analyzed by qPCR and short read sequencing. Then using these two values, the value at each position code was calculated. (**C**) Estimated ChIP recovery of each CenU position codes of the HAC 1-20 cell line. ChIP with CENP-A antibody was carried out, and CenU position codes in each

ChIPed DNA were sequenced and used the results to estimate recovery for each code.

Figure S3

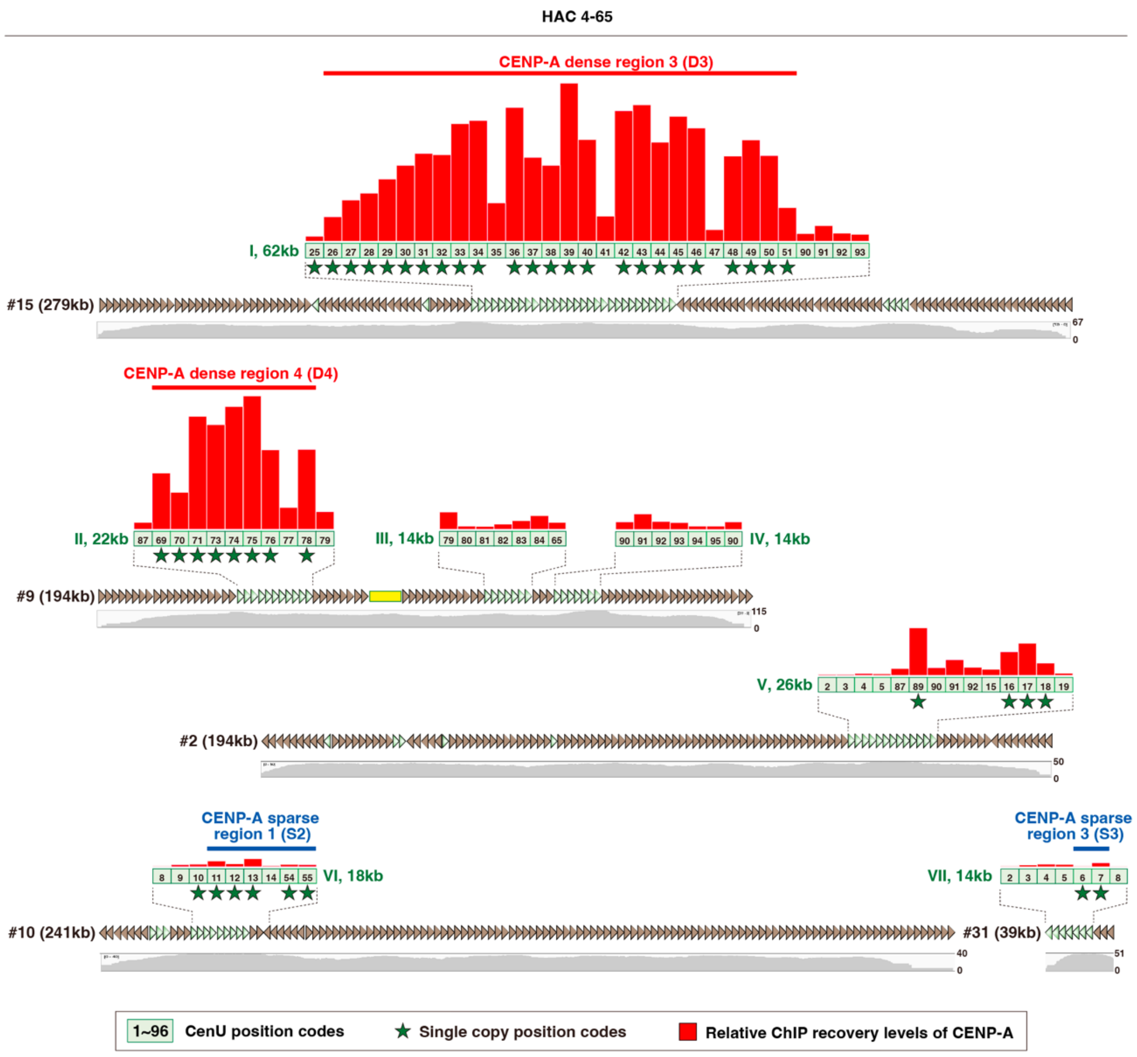

Supplementary Figure S3: Decoding synthetic centromere DNA of HAC 4-65 cell line. Example of contigs containing CenU clusters obtained from long read sequencing of HAC 4-65 cell line. Clusters, which contained more than 5 CenU position code, are shown (I~VII). Numeric number indicate number of each position code. The numbers in green boxes (1~96) indicate the position codes of CenU DNA. Yellow box indicates BAC vector and KM/Neo marker gene cassette. Green stars indicate that the position code above is a single copy. The gray histograms show the coverage of HiFi reads. Red bars, relative CENP-A ChIP recovery level based on Supplementary Figure S2C. CENP-A dense regions are shown as D3 and D4. CENP-A sparse regions are shown as S2 and S3.

Figure S4

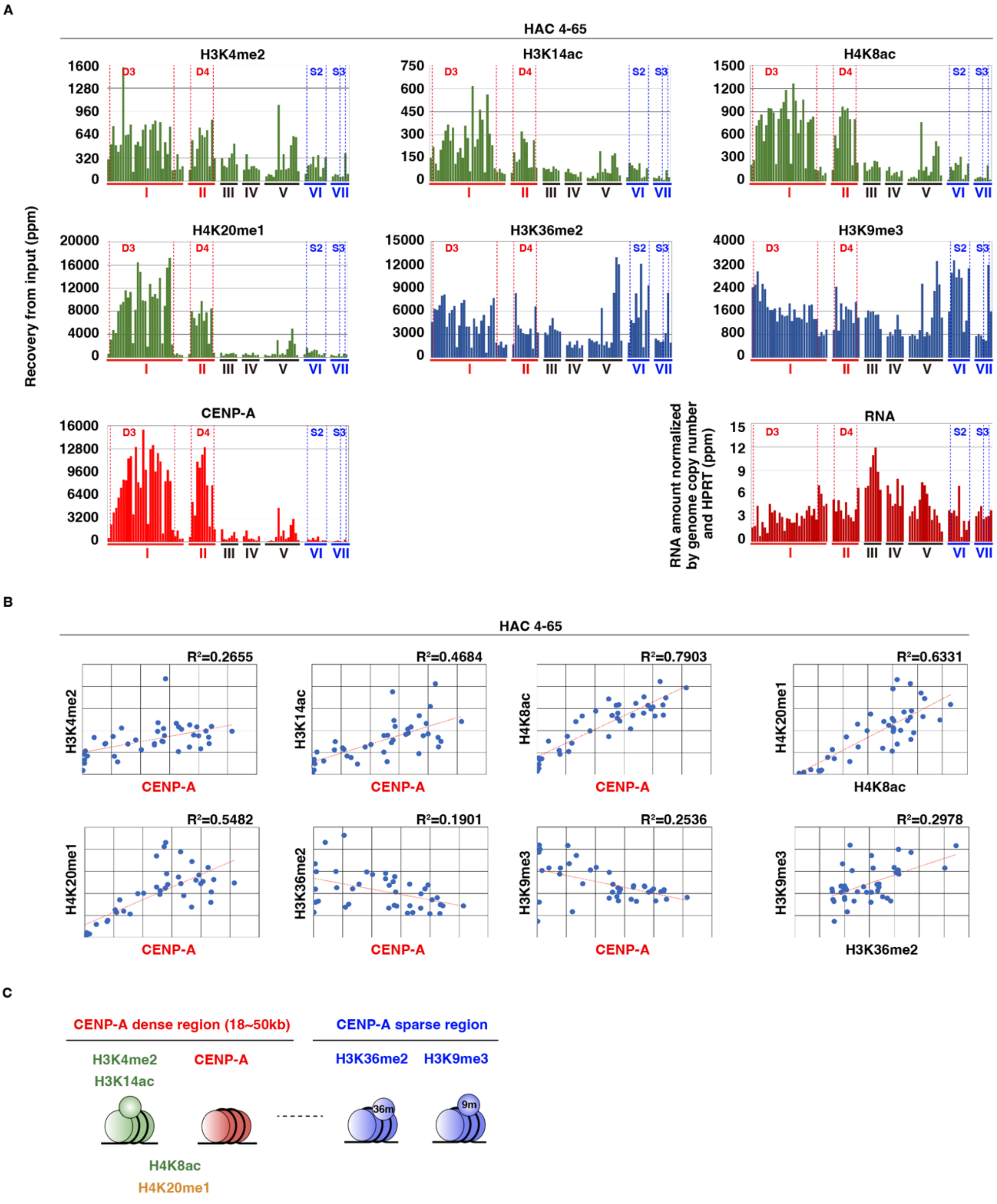

Supplementary Figure S4: Histone modifications distribution of the HAC 4-65 cell line.

(**A**) Estimated ChIP recovery of each CenU position codes of the HAC 4-65 cell line. CenU position codes in each ChIPed DNA were sequenced and used the results to estimate recovery for each code (See also Supplementary Figure S2). CENP-A dense (D3, D4) and sparse (S2,

S3) regions are shown in the graph. (**B**) Correlation plot of each position codes. Pearson's correlation coefficients between CENP-A and each histone modification distribution among the position codes (R^2) are displayed on the right shoulder of each graph. (**C**) Summary of histone modifications in CENP-A dense and sparse regions. Sinse CENP-A nucleosomes do not contain histone H3, there are no H3 modifications with CENP-A nucleosomes, but modifications on histone H4 may be coexist.

Figure S5

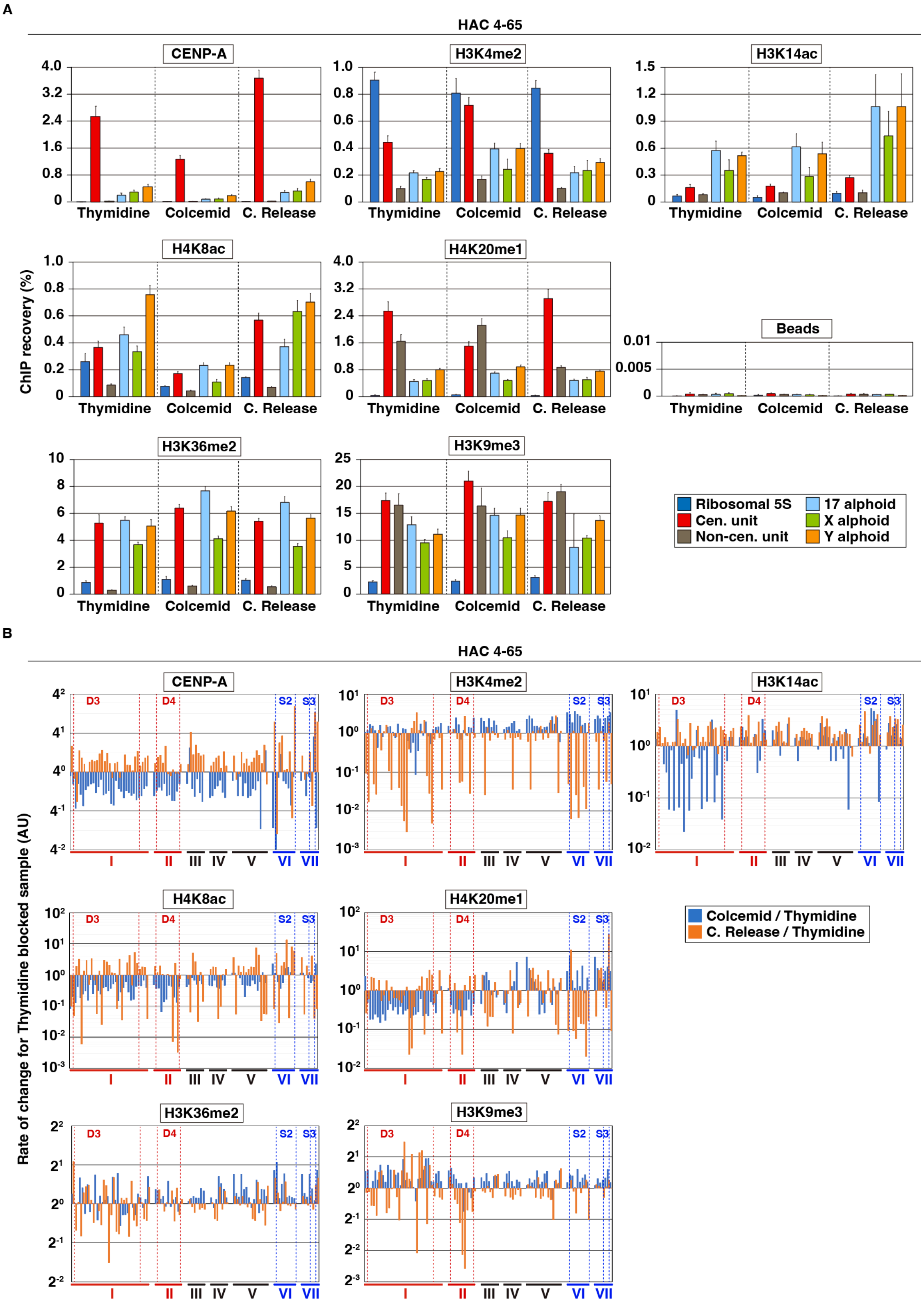

Supplementary Figure S5: Cell cycle synchronized ChIP analysis using HAC 4-65 cells.

(**A**) Cell cycle synchronized ChIP-qPCR analysis of the HAC 4-65 cell lines. ChIP assay was carried out using the synchronized sample sets shown in Figure 5A with indicated antibodies and beads alone. Recovered DNA was quantified by qPCR. Error bar, SD (n=3). (**B**) Rate of change in ChIP recovery relative to thymidine treated G1/S samples. CenU position codes in each ChIPed DNA were sequenced and used the results to estimate recovery for each code. Values from each sample were normalized to the value from the thymidine-treated sample. CENP-A dense (D3, D4) and sparse (S2, S3) regions are shown in the graph.

Figure S6

A

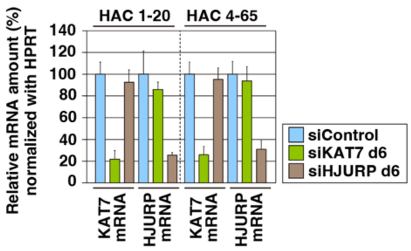

B

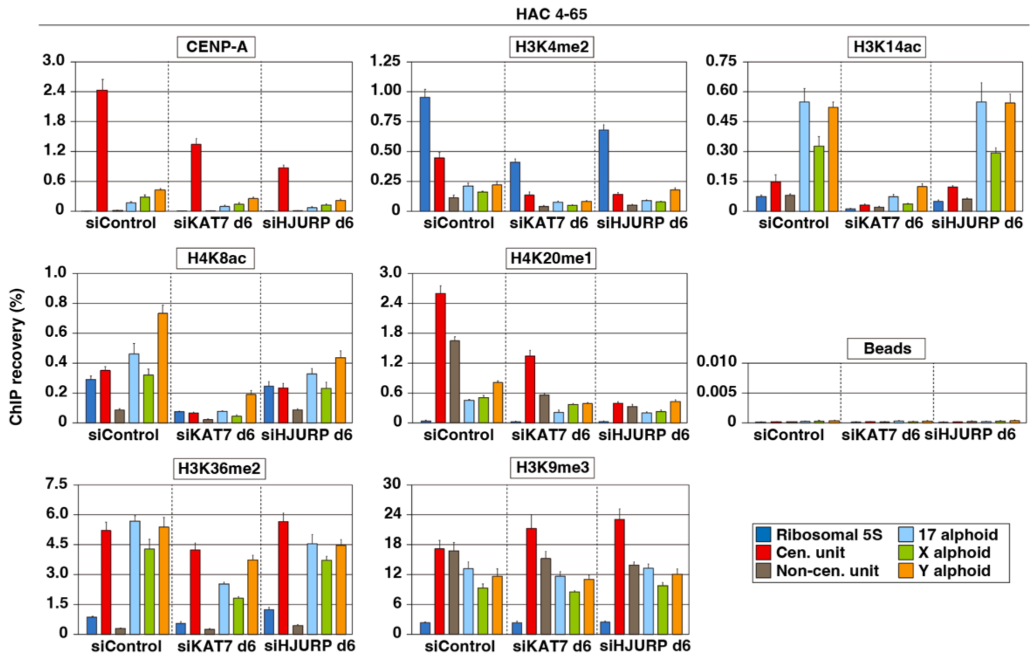

C

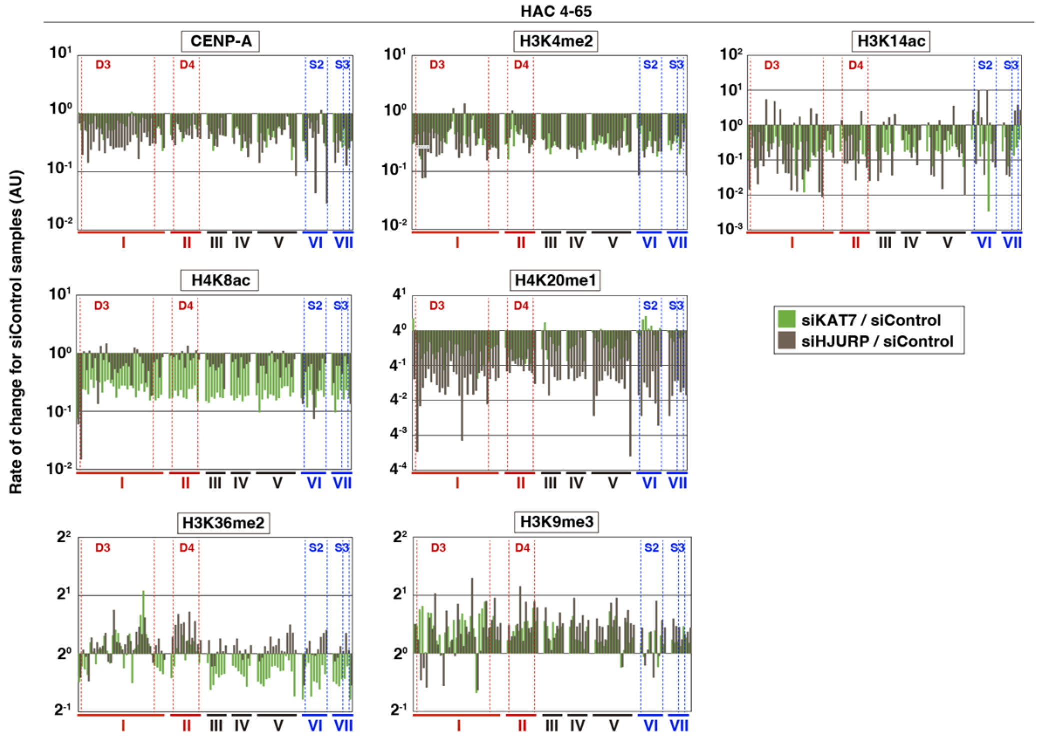

Supplementary Figure S6: KAT7 or HJURP knockdown experiment in the HAC 4-65 cells.
(**A**) Relative mRNA amounts normalized with HPRT. The mRNA level of each gene was also normalized with siControl. Error bar, SD (n=3). (**B**) ChIP-qPCR analysis using mRNA knockdown in the HAC 4-65 cells. ChIP assay was carried out using the siRNA transfected cells shown in Figure 6A with indicated antibodies and beads alone. Recovered DNA was quantified by qPCR. Error bar, SD (n=3). (**C**) Rate of change in ChIP recovery relative to the siControl sample. CenU position codes in each ChIPed DNA were sequenced and used the results to estimate recovery for each code. Values from each sample were normalized to the value from the siControl sample. CENP-A dense (D3, D4) and sparse (S2, S3) regions are shown in the graph.
